# Molecular architecture of the Dam1 complex–microtubule interaction

**DOI:** 10.1098/rsob.150237

**Published:** 2016-03-09

**Authors:** Thibault Legal, Juan Zou, Alicja Sochaj, Juri Rappsilber, Julie P. I. Welburn

**Affiliations:** 1 Wellcome Trust Centre for Cell Biology, School of Biological Sciences, University of Edinburgh, Edinburgh EH9 3BF, UK; 2 Department of Bioanalytics, Institute of Biotechnology, Technische Universität Berlin, Berlin, Germany

**Keywords:** mitosis, Dam1 complex, three-dimensional MS cross-linking, kinetochore, microtubule, self-assembly

## Abstract

Mitosis is a highly regulated process that allows the equal distribution of the genetic material to the daughter cells. Chromosome segregation requires the formation of a bipolar mitotic spindle and assembly of a multi-protein structure termed the kinetochore to mediate attachments between condensed chromosomes and spindle microtubules. In budding yeast*,* a single microtubule attaches to each kinetochore, necessitating robustness and processivity of this kinetochore–microtubule attachment. The yeast kinetochore-localized Dam1 complex forms a direct interaction with the spindle microtubule. *In vitro*, the Dam1 complex assembles as a ring around microtubules and couples microtubule depolymerization with cargo movement. However, the subunit organization within the Dam1 complex, its higher-order oligomerization and how it interacts with microtubules remain under debate. Here, we used chemical cross-linking and mass spectrometry to define the architecture and subunit organization of the Dam1 complex. This work reveals that both the C termini of Duo1 and Dam1 subunits interact with the microtubule and are critical for microtubule binding of the Dam1 complex, placing Duo1 and Dam1 on the inside of the ring structure. Integrating this information with available structural data, we provide a coherent model for how the Dam1 complex self-assembles around microtubules.

## 1. Introduction

Chromosomes must form bioriented attachments to the mitotic spindle to ensure equal partitioning of the genetic material during mitosis. Kinetochores are large macromolecular assemblies that form at centromeres to tether and couple the chromosomes to the plus ends of kinetochore microtubules. At the outer kinetochore, the conserved Ndc80 complex mediates a direct interaction with microtubules. However, the outer kinetochore must also harness the chemical energy released by depolymerizing microtubules and convert it to mechanical energy to move chromosomes. In budding yeast, the 10-subunit Dam1 complex localizes to the outer kinetochore where it attaches to the single incoming microtubule to facilitate chromosome segregation [1]. The Dam1 complex is also a major target of the Aurora B kinase Ipl1 to correct erroneous kinetochore–microtubule attachments [2]. *In vitro* studies have shown that the Dam1 complex assembles as a ring around microtubules [3,4]. This unique property enables the Dam1 complex to processively track the growing and shrinking ends of microtubules under load [5–7]. Thus, the Dam1 complex ring is an excellent candidate to couple microtubule depolymerization with chromosome movement [3,4,8].

Recently, rings or partial rings have been observed at the budding yeast kinetochore [9]. In addition, indirect data from quantitative fluorescence microscopy estimated 16–23 molecules of Dam1 at a single kinetochore, which is compatible with Dam1 rings assembled *in vitro* [10,11]. Low-resolution structures of the Dam1 complex have provided conflicting models for its self-assembly and organization around microtubules, depending on the fitting of the monomeric Dam1 complex [11–13]. Reconstructions of the monomeric Dam1 complex reveal it forms an elongated structure with a protrusion perpendicular to the main axis, that contains the C terminus of Dam1 [11]. In addition, the locations of the N termini of four Dam1 complex subunits and the C terminus of Dam1 itself have been mapped [12]. However, in the absence of high-resolution information, it is not possible to define the structural organization of the Dam1 complex alone, its self-assembly, or its interaction with microtubules. Hence, the subunits and regions of the Dam1 complex that interact with microtubules and their arrangement within the complex remain unknown [11,12]. Here, we used cross-linking mass spectrometry to determine the molecular architecture of the Dam1 complex alone and bound to microtubules. Our data provide a map of the subunit arrangement of the Dam1 complex. Analysis of the Dam1 complex assembled around microtubules reveals that the Spc34 and Ask1 subunits are likely to be involved in self-assembly, whereas the Duo1 and Dam1 subunits both interact with microtubules. We also demonstrate that the C termini of Dam1 and Duo1 provide the microtubule-binding properties to the Dam1 complex. Our data provide key information on the organization of the Dam1 complex around microtubules.

## 2. Results and discussion

### 2.1. Architecture of the Dam1 complex in solution

**Figure 1.**
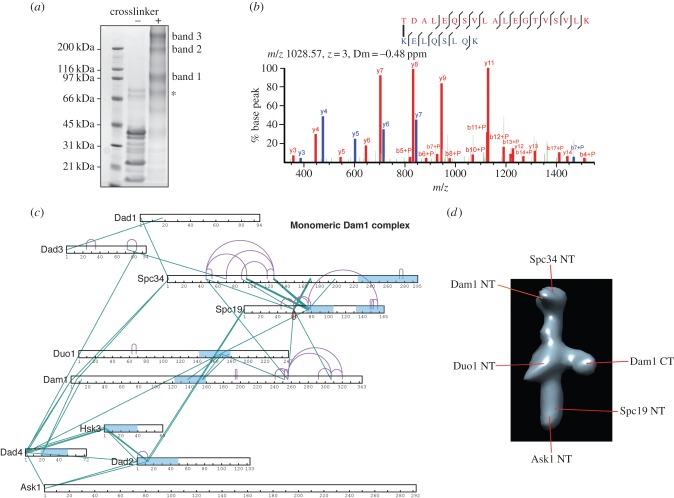
Isolation and architecture of monomeric Dam1 complex by cross-linking. (*a*) SDS–PAGE analysis of the Dam1 complex cross-linked with BS3. Cross-linked Dam1 complex was excised and analysed by mass spectrometry. Asterisk denotes that excluded from our analysis. (*b*) Fragmentation spectrum of a cross-linked peptide that includes a link between Lys-13 in Dad2 and Thr-2 in Spc19. Peaks supporting TDALEQSVLALEGTVSVLK are annotated in red, and those supporting KELQSLQK are annotated in blue. (*c*) Intersubunit and intramolecular cross-link maps for the monomeric Dam1 complex. Intramolecular and intermolecular cross-links are coloured purple and green, respectively. Predicted coiled coil regions are in light blue. (*d*) Representative EM map of the monomeric Dam1 complex (modified from EMDB: 1972), highlighting the reported positions of subunit termini in previous studies.

In solution, the Dam1 complex is predominantly monomeric or dimeric at low concentrations. It self-assembles in oligomeric complexes in a concentration-dependent manner and when it binds to microtubules [3,4]. To define the arrangement of the Dam1 complex, we conducted cross-linking of heterodecameric Dam1 complexes by incubating the label-free bis (sulfo-succinimidyl) suberate (BS3) cross-linker with the Dam1 complex. For these studies, we used low concentrations (0.05 mg ml^−1^) of Dam1 complex such that it forms predominantly monomers and dimers [11]. BS3 reacts with primary amines found at the N termini of proteins and with lysine side chains (and less favourably serine, threonine and tyrosine side chains) with a range of up to 11.4 Å corresponding to the maximal length of the BS3 spacer. This provides a distance from backbone to backbone carbonyl of 27 Å. The cross-linked samples were then separated by SDS–PAGE into three higher-molecular weight species with weights of approximately 110, 220 and 440 kDa (figure 1*a*). Using mass spectrometry, we first identified the composition of each band and the cross-links within each sample (figure 1*b*). The bands corresponding to the 220 and 440 kDa species both contained all 10 subunits of the Dam1 complex, whereas the 110 kDa species contained a seven protein complex lacking Dam1, Duo1 and Dad1 subunits (electronic supplementary material, figure S1 and table S1). The isolation of this Dam1 subcomplex is most likely due to incomplete cross-linking, followed by subsequent separation of the proteins by denaturing SDS–PAGE. These cross-linking data suggest these seven proteins form a core complex, and that Dam1, Duo1 and Dad1 may be associated with the rest of the complex through a smaller surface. Strikingly, this identified complex is very similar to that formed in the absence of Dam1 [10]. We did not pursue the Dam1 subcomplex species further.

To define the molecular arrangement of the various subunits within the Dam1 complex, we analysed the monomeric and dimeric Dam1 complexes from the 220 and 440 kD species by mass spectrometry (electronic supplementary material, tables S2 and S3). For each sample, we identified all 10 protein subunits. For the monomeric Dam1 complex, we could detect subgroups and intramolecular interactions emerging within the complex (figure 1*c*). The C-terminal regions of Dam1 and Duo1 displayed long-range cross-links internally and with each other, highlighting that these domains share a strong interface and suggesting they may have a globular subdomain conformation. Our data reveal that the central coiled coil region of Dam1 (125–158) assembles in a coiled coil with Duo1 (152–180). This could explain why in the absence of Dam1, Duo1 is not found in the remaining complex [10].

Notably, the N terminus of Ask1 formed cross-links with the N termini of Dad4 and Dad2. Thus, Ask1–Dad4–Dad2 and Hsk3 probably form the lower part of the Dam1 complex, with the N terminus of Ask1 at the most terminal part, as mapped in previous electron microscopy (EM) studies (figure 1*d*) [12]. We did not obtain many internal cross-links for Dad1, Dad2, Dad3, Dad4 and Hsk3, in part owing to their small size. The N termini of the Dad2, Dad4 and Hsk3 subunits containing α-helical coiled coils formed multiple cross-links, suggesting tight interactions between them (figure 1*c*). Based on their high α-helical content and our cross-linking data, Dad2, Dad4 and Hsk3 do not form globular structures, but instead assemble in a coiled coil. The N terminus of Spc34, previously mapped in the Dam1 monomer by EM, formed cross-links with the N terminus of Dam1 [12]. Thus, our data indicate that the N terminus of Dam1 is close to the N terminus of Spc34 (figure 1*d*). The middle part of Spc34 also cross-linked with Spc19, confirming previous yeast-two hybrid interaction studies [14].

**Figure 2.**
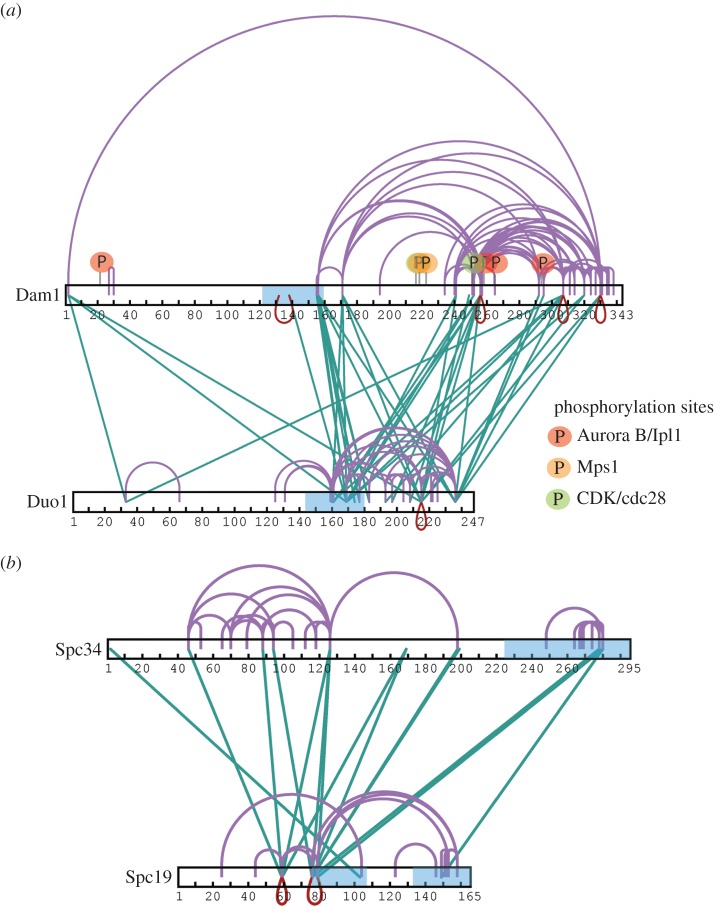
Duo1/Dam1 and Spc34/Sp19 form tight interfaces within the Dam1 complex. Cross-link maps for (*a*) Duo1 and Dam1 and (*b*) Spc19 and Spc34 show intramolecular and intermolecular cross-links, isolated from in-solution digestion of the Dam1 complex. Green cross-links represent intermolecular links. Red and purple connections represent intermolecular self-links (between homodimers) and intermolecular or intramolecular self-cross-links, respectively. Predicted coiled coil regions are highlighted in blue. Regulatory sites within Dam1 targeted by Cdc28/CDK1 (S216, S250), Ipl1 (S20, S257, S265, S292) and Mps1 (S218 and S221) are highlighted in green, red and orange, respectively.

After cross-linking at low concentration, in-gel digestion and extraction of the cross-linked Dam1 complex, the yield of cross-links was low. This low recovery is due to the small amount of cross-linked proteins added to each well and extracted from each band, the low peptide recovery from in-gel digestion, and the general decreased efficiency of the cross-linker at low protein concentration. To increase our density of cross-links and the resolution of the architecture of the complex, we next cross-linked a higher concentration of Dam1 complex (0.3 mg ml^−1^) and digested the sample in solution rather than following gel extraction. This considerably improved our yield of cross-links, especially in regions for which we already had cross-links, thereby increasing the data for these interactions (figure 2 and electronic supplementary material, table S4). However, some cross-links may be from connections formed between oligomerizing Dam1 complexes, given that at concentrations around 0.5 mg ml^−1^ the Dam1 complex self-assembles into rings in the absence of microtubules [12].

Dam1 is one of the largest proteins in the decameric complex and occupies a central position in the complex. From our data, the N terminus of Dam1 is at the heart of a tight interaction network, with the N-terminal regions of Dad2–Hsk3–Dad4–Spc34 forming a strong network around the N terminus of Dam1, possibly resulting from oligomerization (electronic supplementary materials, table S4). This may explain why epitope tagging of the N terminus of Dam1 has proved impossible, and why expression of the nine-protein complex lacking Dam1 results in fragmentation of the complex [10,12]. Interestingly, the C-terminal region of Dam1 shares a large interaction surface with the C terminus of Duo1, displaying extensive cross-links both at low and high cross-linking concentration, in the region containing residues that are phosphorylated by Ipl1 (figures 1*c* and 2*a*) [2]. The C terminus of Dam1 forms the outer arm of the Dam1 complex (figure 1*d*) [11]. Our data suggest the C terminus of Duo1 is also part of this outer arm.

At both low and high concentration of the Dam1 complex during cross-linking, we identified extensive cross-links between the two α-helical coiled coils of Spc19 (73–104 and 132–165), suggesting that they form an anti-parallel dimer (figure 2*b* and electronic supplementary material, tables S1 and S4). There were also multiple cross-links between Spc19 and Spc34, revealing a strong interface between these two proteins. At high concentration, we also obtained many cross-links between the C termini of Duo1 and Dam1 and specifically the N terminus of Ask1 and the C termini of Spc34 and Spc19 (electronic supplementary material, table S4). In the monomeric Dam1 complex and from the subunit mapping from the EM studies, Ask1 and Spc34/Spc19 are spatially separated [12]. Therefore, these data suggest that Ask1 and Spc34 may be the regions involved in the Dam1 complex oligomerization interface. In total, our data provide a physical interaction map for the Dam1 complex and reveal the architecture and organization of the Dam1 complex into key protein subcomplexes.

### 2.2. The Duo1 and Dam1 subunits interact directly with microtubule polymers

**Figure 3.**
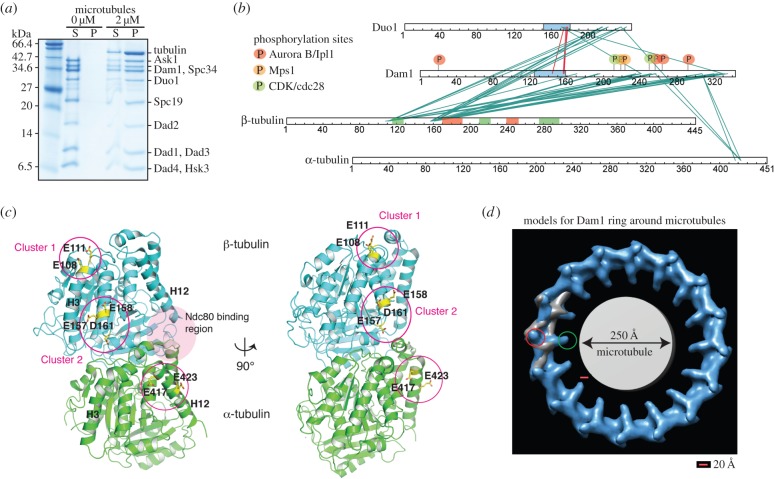
The Dam1 complex interacts with microtubules by recognizing the globular regions of tubulin monomers. (*a*) Coomassie-stained gel shows cosedimentation of the Dam1 complex in the presence of 2 µM taxol-stabilized microtubules. (*b*) Linkage map shows the sequence position of all the cross-linked residue pairs between the Dam1 complex and porcine α- and β-tubulin. Cross-linked products were digested in solution followed by MS analysis. Red and green boxes above the β-tubulin show regions of tubulin involved in longitudinal and lateral contacts, respectively. Predicted coiled coil regions are in blue. Regulatory sites within Dam1 targeted by Cdc28/CDK1 (S216, S250), Ipl1 (S20, S257, S265, S292) and Mps1 (S218 and S221) are highlighted in green, red and orange respectively. (*c*) Cartoon representation of tubulin dimer where residues involved in cross-linking with the Dam1 complex residues are highlighted in stick representation. The region where Ndc80 interacts with tubulin as reported in is shown in pink [15]. (*d*) Proposed models for the assembly of the Dam1 complex around the microtubules. Model of the Dam1 complex assembled as a ring (right, EMDB: 5254 [13]). The map of the Dam1 complex dimer (EMDB: 1972) is fitted into the electron density map of the Dam1 complex assembled as a ring and is painted in grey. The location of the C terminus of Dam1 in this model is highlighted as a red circle. The location of the C terminus of Dam1 mapped from the earlier proposed model (EMDB: 1371) is circled in green. Scale bar represents 20 Å.

After defining the direct interaction map between the subunits within the Dam1 complex, we next sought to determine the arrangement and self-assembly mechanism of the Dam1 complex around microtubules. There are two different structural models for the assembly of the Dam1 complex around microtubules, based on the structures of the Dam1 complex alone and bound to microtubules derived by cryoelectron microscopy [11,12]. One model (model 1, EMDB: 1371), obtained from a helical assembly of Dam1 complexes around microtubules, proposes that the C terminus of Dam1 is on the inside of the ring and points towards the microtubule. This is in agreement with previous studies showing Dam1 had its own microtubule-binding activity [3,10]. This model suggests a major conformational rearrangement of the Dam1 complex occurs on binding to microtubules, around the central core of the complex, based on the fitting of the monomeric Dam1 complex into the Dam1 helical reconstruction around microtubules [11]. The second model, derived from a single particle reconstruction of individual rings of assembled Dam1 complexes around microtubules [12,13] (model 2, EMDB: 5254), positions the C-terminal protrusion of Dam1 on the outside of the Dam1 ring, 300 Å away from the microtubule lattice. In this model, the Dam1 complex does not undergo any conformational changes upon oligomerizing on microtubules and the C terminus of the Dam1 complex is at the self-assembly interface. To determine which model is correct and to define which subunits interact with the microtubules, we assembled the Dam1 complex on microtubules (figure 3*a*) and cross-linked the sample with 1-ethyl-3-[3-dimethylaminopropyl] carbodiimide hydrochloride (EDC). EDC cross-links lysine side chains and primary amine groups (and less favourably serine, threonine and tyrosine side chains) to aspartate or glutamate carboxyl groups. Mass spectrometry analysis of the cross-linked products revealed that the C termini of Duo1 and Dam1 both made specific cross-links with α- and β-tubulin (figure 3*b* and electronic supplementary material, table S5) [16]. Surprisingly, the cross-links obtained involved the solvent-exposed folded domains of α- and β-tubulin rather than their acidic tails. Duo1 and Dam1 cross-linked to E417 and E423 in α-tubulin (α-tub_cluster_), and to E108 and E111 (β-tub_cluster1_), and E157, E158 and D161 (β-tub_cluster2_) in β-tubulin (figure 3*c*). These interactions are reminiscent of the binding of the Ska1 complex, thought to be the human orthologue of the Dam1 complex, to microtubules [17]. The termini of Dam1 and Duo1 are thought to be flexible and unstructured [10], unlike the human counterpart Ska1 complex. However, they appear to bind to the same region on the microtubule lattice. Given that the maximum length that EDC can cover is 20 Å, this indicates that the C termini of Duo1 and Dam1 are both within 20 Å of the microtubule (figure 3*d*). Therefore, it is unlikely that the protrusion arm containing Dam1 and Duo1 is on the outside of the ring, as proposed by model 2 [12].

### 2.3. Spc34 and Ask1 interact during self-assembly of the Dam1 complex around microtubules

**Figure 4.**
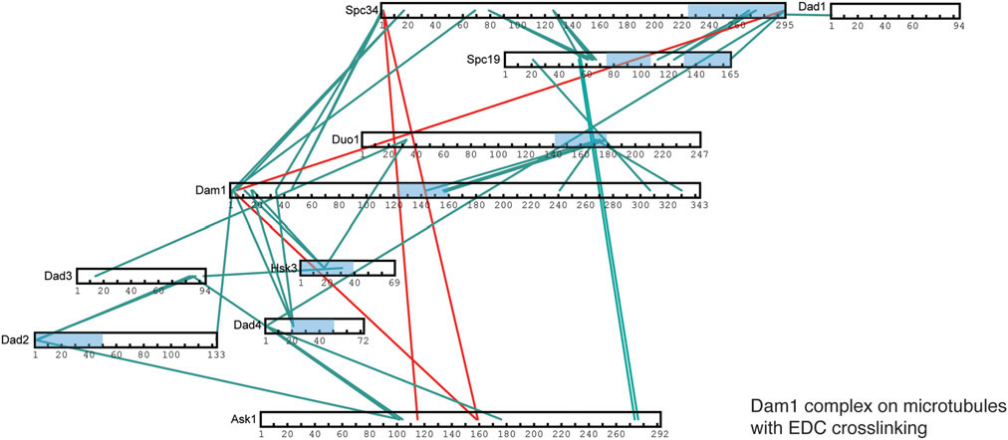
Architecture of the Dam1 complex on microtubules. Cross-links within the Dam1 complex in the presence of EDC. Cross-links present in the Dam1 complex assembled on microtubules, but absent in the monomeric Dam1 complex cross-linked in solution, are highlighted in red. Cross-links present for the Dam1 complex cross-linked at low concentration in solution are highlighted in green. Predicted coiled coil regions are in blue.

To determine how oligomerization of the Dam1 complex around microtubules occurs, we compared the cross-links obtained for the Dam1 complex in solution and when bound to microtubules. We mapped the cross-links that were unique to the Dam1 complex self-assembled around microtubules. From the subunit mapping onto the EM structure of the monomer, Spc34 and Ask1 are at the opposite ends of the monomer [12]. However, in the presence of microtubules, we observed that Ask1 cross-linked to Spc34 with both BS3 and EDC cross-linkers (figure 4 and electronic supplementary material, tables S5 and S6). This indicates that, when the Dam1 complex is oligomerized around microtubules, Ask1 is in close proximity to Spc34 (figure 4). We also found Ask1 cross-links to the N terminus of Dam1, while still cross-linking to Dad2 and Dad4 (figure 4 and electronic supplementary material, table S5). In addition, Spc34 and Spc19 formed cross-links with Ask1 and the N terminus of Dad4 when the Dam1 complex was cross-linked at a concentration at which it oligomerizes (electronic supplementary material, table S4). Therefore, we suggest that Spc34 and Ask1 form intercomplex interfaces during self-assembly of the Dam1 complex around microtubules. Spc34 and Spc19 cross-links were obtained from the Dam1 subcomplex, as well as from cross-linked monomeric and dimeric Dam1 complex (figure 1*c* and electronic supplementary material, figure S1), arguing that these cross-links are intrinsic to the monomeric Dam1 complex, rather than between monomers [16]. In addition, yeast two-hybrid studies showed a direct interaction between the regions of Spc34 and Spc19 for which we obtained cross-links [14]. Finally, at high concentrations of the Dam1 complex, we observed extensive cross-linking of the Dam1 and Duo1 C termini with themselves and with other subunits, in particular the C terminus of Spc34. These cross-links suggest these proteins may promote self-assembly through self-interaction (figure 2*a*). In agreement with this observation, the Dam1 complex lacking the C terminus and the phosphomimetic Dam1 complex with mutations mimicking Ipl1 phosphorylation showed a decreased ability to oligomerize and a reduced affinity for microtubules [11]. However, most of these interactions were not observed when the Dam1 complex was assembled on microtubules. Therefore, Duo1 and Dam1 together play a pivotal role in recruiting the Dam1 complex to microtubules and promoting its self-assembly.

### 2.4. The C terminus of Duo1 is essential for the integrity of the Dam1 complex and for its interaction with microtubules

**Figure 5.**
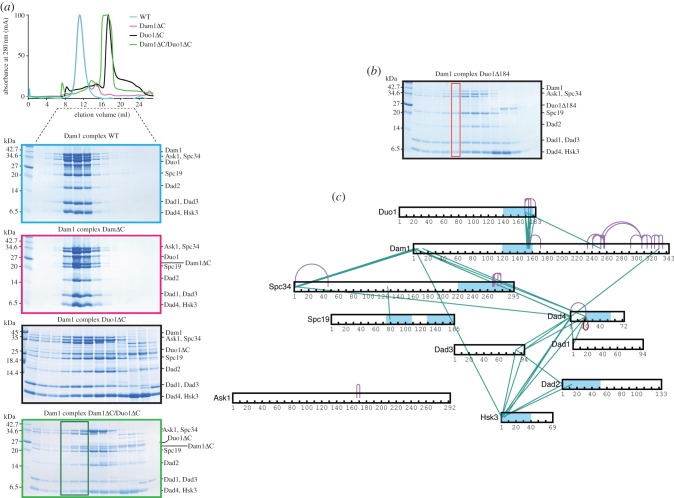
The C terminus of Duo1 is important for the structural integrity of the Dam1 complex. (*a*) Top, gel filtration elution profile of Dam1 complex (light blue), Dam1 complex Dam1ΔC (magenta), Dam1 complex Duo1ΔC (black), and Dam1 complex Dam1ΔC/Duo1ΔC (green). Bottom, protein gels showing the size-exclusion chromatography profile for each Dam1 complex mutant. Fractions boxed in dark green were tested in microtubule co-sedimentation assays in figure 6. (*b*) Protein gel showing the size-exclusion chromatography profile for the Dam1 complex Duo1Δ184. The fraction boxed in red was used for cross-linking and mass spectrometry analysis in (*c*). (*c*) Cross-links within the Dam1 complex Duo1Δ184 in the presence of BS3. Predicted coiled coil regions are in blue.

To test the role of the C termini of Duo1 and Dam1 on the assembly and microtubule-binding properties of the Dam1 complex, we generated mutants of the Dam1 complex lacking the C terminus of Duo1 (Δ184 and Δ211) and both the C termini of Duo1 and Dam1. We first purified the Dam1 complex *Dam1–19*, which lacks the last 138 amino acids of the C terminus (Dam1ΔC). It behaved similarly to the Dam1 complex by analytical size-exclusion chromatography (figure 5*a*) [3]. We then purified the Dam1 complex Duo1Δ184 and Dam1 complex Duo1Δ211, further termed Duo1ΔC (figure 5*a*,*b*). All 10 proteins could still be purified using the affinity purification tag on Hsk3 and identified by mass spectrometry. Unexpectedly, we could purify the complex of 10 proteins but also obtained some smaller complexes containing Hsk3 for both mutants (figure 5*a*,*b*). To further dissect the architecture of the Dam1 complex lacking the C terminus of Duo1, we conducted cross-linking on a fraction corresponding to the elution of the full Dam1 complex Duo1Δ184 (red box; figure 5*b*). We obtained multiple cross-links between the coiled coil regions of Dam1 and Duo1, as previously (figure 5*c* and electronic supplementary material, table S7). We still observed a tight network of cross-links between Dad1, Dad2, Dad3, Dad4 and Hsk3. However, we did not find many cross-links between the other subunits, when compared with wild-type Dam1 complex. Thus, while all 10 proteins co-eluted as a complex, the structural integrity of the Dam1 complex was affected by the absence of Duo1 C terminus. Interestingly, Zelter *et al.* [16] found the C terminus of Duo1 to cross-link with Dad1, Dad2, Dad3, Spc19 and Spc34 in the absence of microtubules, which agrees with the role of Duo1 we reveal presently.

**Figure 6.**
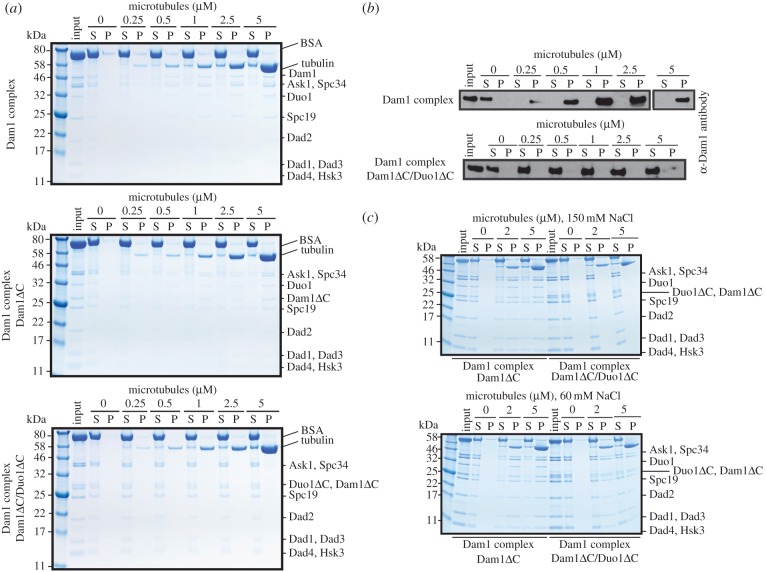
Both the C termini of Duo1 and Dam1 are necessary for the association of the Dam1 complex with microtubules. (*a*) Protein gel shows the cosedimentation of 350 nM Dam1 complex, Dam1 complex Dam1ΔC and Dam1 complex Dam1ΔC/Duo1ΔC at the indicated concentration of microtubules. BSA is present in the supernatant to reduce non-specific binding. (*b*) Western blot shows the cosedimentation of 350 nM Dam1 complex and Dam1 complex Dam1ΔC/Duo1ΔC at the indicated concentration of microtubules. (*c*) Protein gels show the cosedimentation of 1.5 µM Dam1 complex Dam1ΔC and Dam1 complex Dam1ΔC/Duo1ΔC at the indicated concentration of microtubules in the presence of 150 and 60 mM NaCl.

Our cross-linking data suggested Duo1 and Dam1 were the two subunits contributing to the microtubule-binding properties of the Dam1 complex. To further test this, we purified a Dam1 complex Dam1ΔC/Duo1ΔC, lacking the C termini of Duo1 and Dam1. Similar to the Dam1 complex Duo1ΔC mutants, we also recovered smaller subcomplexes containing his-tagged Hsk3, which could be separated by size-exclusion chromatography (figure 5*a*). We then tested the microtubule-binding properties of the Dam1 complex Dam1ΔC/Duo1ΔC eluting as a 10-protein complex (dark green box) using 350 nM Dam1 complex in a microtubule co-sedimentation assay. The Dam1 complex Dam1ΔC/Duo1ΔC remained in the supernatant in the presence of microtubules, whereas the Dam1 complex, the Dam1 complex Duo1ΔC and the Dam1 complex Dam1ΔC cosedimented with microtubules, as previously shown (figure 6*a*,*b* and electronic supplementary material, figure S2) [3]. Thus, we demonstrated that the Dam1 complex lacking both the C termini of Dam1 and Duo1 no longer has the ability to bind to microtubules. In lower ionic strength (60 mM NaCl), we observed weak binding of the Dam1 complex Dam1ΔC/Duo1ΔC to microtubules. However, the Dam1 complex Dam1ΔC/Duo1ΔC bound less to microtubules than the Dam1 complex Dam1ΔC, which could still robustly bind to microtubules (figure 6*c*). Thus, these data indicate that the C terminus of Duo1 contributes to the binding of the Dam1 complex to microtubules. The residual binding of the Dam1 complex Dam1ΔC/Duo1ΔC to microtubules is most likely due to both the presence of residues 160–205 in Dam1, which interact with microtubules electrostatically (figure 3*b*), and the cooperativity of the Dam1 complex to self-assemble on the microtubules, even when the affinity of a single Dam1 complex for microtubules is very low. Taken together, the C terminus of Duo1 forms the core of the interaction network within the Dam1 complex, is essential for the stability of the Dam1 complex in solution and is a major contributor to the microtubule-binding properties of the Dam1 complex.

### 2.5. Duo1 and Dam1 as force-transducing couplers during chromosome segregation

Structural insights into the Dam1 complex have been generated previously from a low-resolution map of the monomeric Dam1 complex combined with the positioning of the N termini of Spc34, Duo1, Ask1 and Spc19 and the C terminus of Dam1 in this EM density [12]. Using an orthogonal approach, we now report a structural map of the Dam1 complex that provides new structural insights, advancing our understanding of the Dam1 complex connectivity and organization. Our data are also in good agreement with previous yeast two-hybrid studies of the Dam1 complex [14]. In addition, we show unambiguously that the C termini of Dam1 and Duo1 are the two contributors to the interaction of the Dam1 complex with microtubules [16]. Dam1 and Duo1 bind to multiple sites at the surface of the microtubule lattice. These results explain why in the absence of the C terminus of Dam1 (in a *dam1–19* temperature sensitive mutant), the Dam1 complex is still functional such that the yeast are able to grow, but have very short spindles [1]. The *dam1–19* mutant complex has a reduced, but not absent, affinity for microtubules and its force-transducing coupling ability is reduced [8]. Thus, the C terminus of Duo1 still provides microtubule-anchoring function to the Dam1 complex, even in the absence of the Dam1 C terminus.

The acidic tail of tubulin increases the strength of the Dam1 complex–microtubule interaction, but is not the major determinant of this interaction. Phosphorylation of the C terminus of Dam1 by Ipl1 reduces the affinity of the Dam1 complex for microtubules *in vitro* and constitutively mimicking this phosphorylation disrupts microtubule interactions *in vivo* [2,3]. The large number of cross-links between the lysine residues in the C terminus of Dam1 and the acidic residues in tubulin indicate that the interaction is electrostatic and that phosphorylation reduces this interaction through electrostatic repulsion. The fungal Dam1 complex and the metazoan Ska1 complex are structurally distinct, but are likely to represent functional homologues [18]. Interestingly, both the Dam1 and Ska1 complexes bind to the microtubule using a similar interface with tubulin rather than the acidic tails of tubulin [17]. The Ska1 complex binds both straight and curved microtubule protofilaments [19]. The Dam1 complex may also bind to curved microtubules, according to the conformational wave model [20]. Our data highlight that the Dam1 complex uses the acidic tails of tubulin to enhance its affinity for the microtubules, but primarily recognizes the microtubule surface. The acidic tails of tubulin may help with electrostatic lattice diffusion of the Dam1 complex, similarly to MCAK [21,22]. Our work and proteolytic experiments on the Dam1 complex associated with the microtubule show that the C termini of Dam1 and Duo1 are both flexible and necessary for microtubule binding [10]. In addition, the electron density for the Dam1 complex close to microtubules is very weak and averaged out during refinement, which is typical for flexible regions [10–12]. Taken together, the C termini of Dam1 and Duo1 are flexible and Duo1 and Dam1 interact with the microtubule lattice electrostatically rather than making a footprint on the lattice.

Our data and Zelter *et al*. [16] also suggest that the C terminus of Duo1 is involved in an important conformational change during the solution to microtubule-binding transition. Although our data cannot determine the extent of the conformational change upon binding of the Dam1 complex to microtubules proposed by the structural model 1, we can rule out the structural model 2 for self-assembly of the Dam1 complex around microtubules based on the close proximity of the C termini of Dam1 and Duo1 to microtubules within 20 Å (figure 3*d*) [16]. The site occupied by the Dam1 complex on the lattice is also compatible with Ndc80 complex binding at the interface between the α-tubulin and β-tubulin (figure 3*c*) [15,23]. Finally, our work suggests we can uncouple the microtubule-binding properties of the Dam1 complex from its oligomerization properties (figure 6). Future high-resolution work is required to understand how the Dam1 complex oligomerizes around microtubules to couple chromosome movement to depolymerizing microtubules.

## 3. Material and methods

### 3.1. Dam1 complex protein purification and cosedimentation assay

The Dam1 complex was expressed and purified as described [3]. Site-directed mutagenesis of Duo1 was performed using DNA oligos for Duo1Δ184 5′-gatttaagccttgatttggttactttgctggtgcagcatccttt-3′ and 5′-aaaggatgctgcaccagcaaagtaaccaaatcaaggcttaaatc-3′ and for Duo1Δ211 5′-atgggtctttcttactcttcagttatttgaaattcctgccg-3′ and 5′-cggcaggaatttcaaataactgaagagtaagaaagacccat-3′ to insert a stop codon at position 184 and 211, respectively. The Dam1 complexes Duo1ΔC and Dam1ΔC/Duo1ΔC were purified using Ni–NTA affinity purification (GE Healthcare) followed by size-exclusion chromatography on a Superose 6 column (GE Healthcare), as they did not bind to the ion-exchange column. Microtubule cosedimentation assay was performed as previously described [22]. The binding assay was performed in a final concentration of 150 mM NaCl, unless stated otherwise. The samples were resolved by SDS–PAGE (12% Bis–Tris or Tricine 16% NuPAGE, Invitrogen) and stained using Instant Blue (Expedeon). For Western blotting, the proteins were transferred to a nitrocellulose membrane and probed for Dam1 using a rabbit anti-Dam1 antibody (kind gift from Prof. Tomo Tanaka). Experiments were repeated three times.

### 3.2. Protein cross-linking

The mixing ratio of BS3 (Thermo Fisher Scientific) to complex was determined for the Dam1 complex using 5 mg aliquots and using a protein-to-cross-linker ratio (w/w) of 1 : 1, 1 : 2, 1 : 3, 1 : 4, 1 : 5, 1 : 6 and 1 : 7, respectively. Low concentration of the Dam1 complex, at 0.05 mg ml^−1^, was used to minimize cross-linking of higher-order structures. As the best condition, we chose the ratio that was sufficient to convert most of the individual Dam1 subunits into a high molecular weight band corresponding to the monomeric and dimeric Dam1 complexes (1 : 5–1 : 7), but did not cross-link the Dam1 complex into aggregates, as judged by SDS–PAGE analysis. The reactions were resolved by SDS–PAGE (4–12% Bis–Tris NuPAGE, Invitrogen) gel separation and stained using Instant Blue (Expedeon). The bands were then excised, and the proteins therein were reduced using 5 mM BME for 30 min at room temperature, alkylated with 55 mM iodoacetamide for 20 min in the dark at room temperature and digested using trypsin (sequencing grade; Promega) overnight at 37°C. Cross-linked peptides were analysed as previously described [17].

#### 3.2.1. Preparation of cross-linked Dam1 complex alone (high concentration)

Purified Dam1 complex of 6 µg was mixed with 20 µg BS3 dissolved in 10 µl BRB80 at a final concentration of 0.3 mg ml^−1^ and incubated at room temperature for 40–60 min. The reaction was stopped by adding 2.5 µl of 2.5 M ammonium bicarbonate for 45 min on ice. The protein was then precipitated with 20% TCA and left overnight at 4°C.

#### 3.2.2. Preparation of Dam1 complex cross-linked to microtubules

The purified Dam1 complex (6 µg) was incubated with 4 µg taxol-stabilized microtubules in BRB80 (80 mM PIPES pH 6.80, 1 mM EGTA, 1 mM MgCl_2_) for 10 minutes at room temperature. The complex was then spun at 13 000 r.p.m. at room temperature in a benchtop centrifuge to remove free tubulin and unbound Dam1 complex. The microtubule–Dam1 complex pellet was washed twice in warm BRB80 buffer containing 2 µM taxol. The Dam1–microtubule complex was mixed with 10 µg 1-ethyl-3-[3-dimethylaminopropyl] carbodiimide hydrochloride (EDC, Thermo Fisher Scientific) and 22 µg of *N*-hydroxysulfosuccinimide (Sulfo-NHS, Thermo Fisher Scientific) dissolved in 20 µl BRB80 and incubated at room temperature for 1 h. The complex was then spun at 13 000 r.p.m. at room temperature and lyophilized.

#### 3.2.3. Preparation of samples for mass spectrometry analysis

Cross-linked complex proteins were reduced, alkylated and digested following standard procedures [24]. Trypsin (Promega) and Lys-C (Roche) digestion was then performed according to manufacturer's protocols. Cross-linked peptides were desalted using C18 StageTips [25,26].

### 3.3. Mass spectrometry

Peptides were analysed on LTQ Orbitrap Velos (Thermo Fisher Scientific) as previously described [17].

## Supplementary Material

Supplementary Figure 1

## Supplementary Material

Supplementary Figure 2

## Supplementary Material

Table S1

## Supplementary Material

Table S2

## Supplementary Material

Table S3

## Supplementary Material

Table S4

## Supplementary Material

Table S5

## Supplementary Material

Table S6

## Supplementary Material

Table S7

## Data accessibility

The mass spectrometry proteomics data have been deposited to the ProteomeXchange Consortium via the PRIDE [27] partner repository with the dataset identifier PXD003678.
