# Supplementary figures and images for: Molecular architecture of the Dam1 complex–microtubule interaction
Source: Open Biol. 2016 Mar 9;6(3):150237. doi: 10.1098/rsob.150237 (PMC4821239; doi:10.1098/rsob.150237)

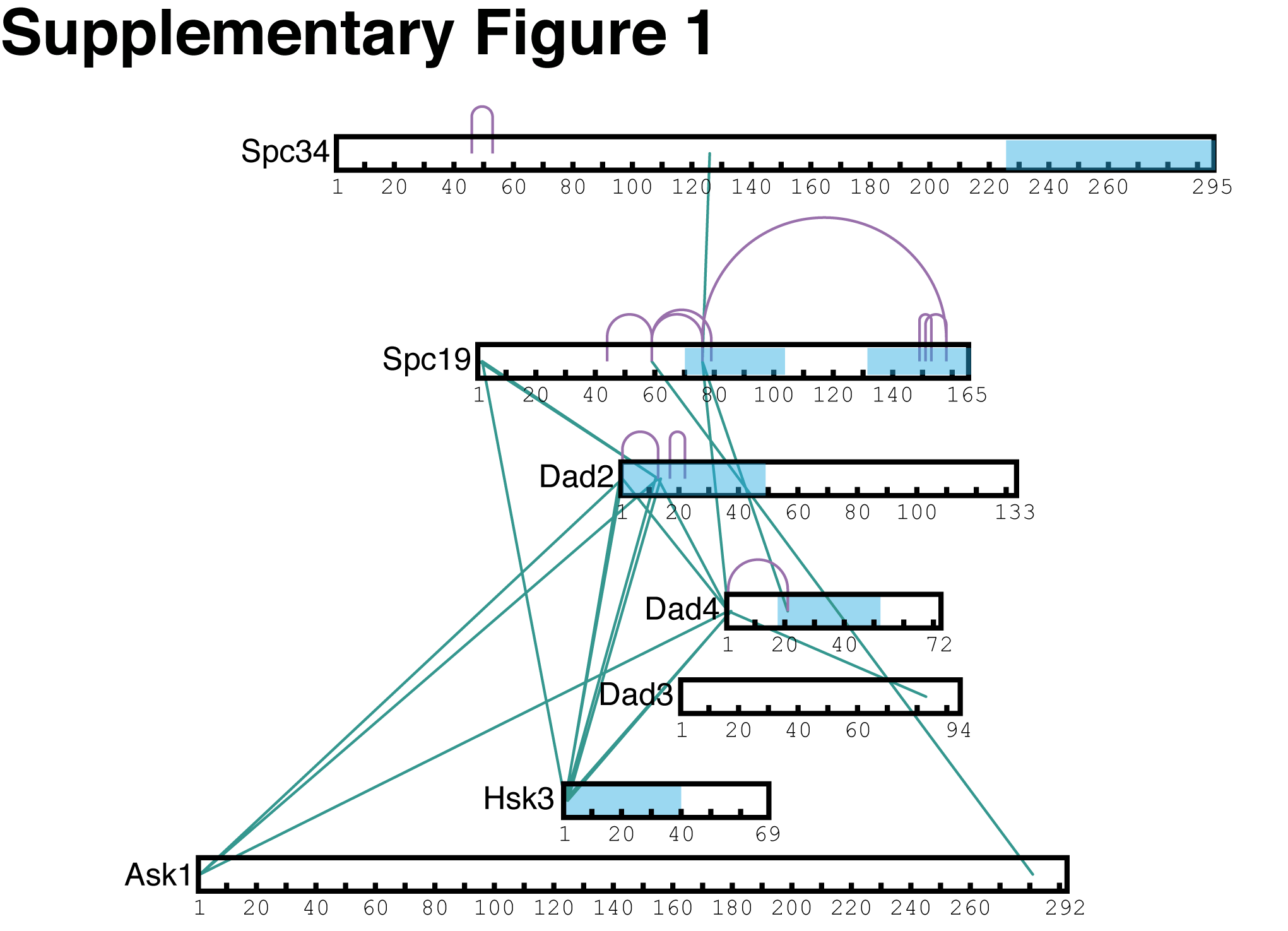

Supplement: Supplementary Figure 1 [file rsob150237supp1.tif]

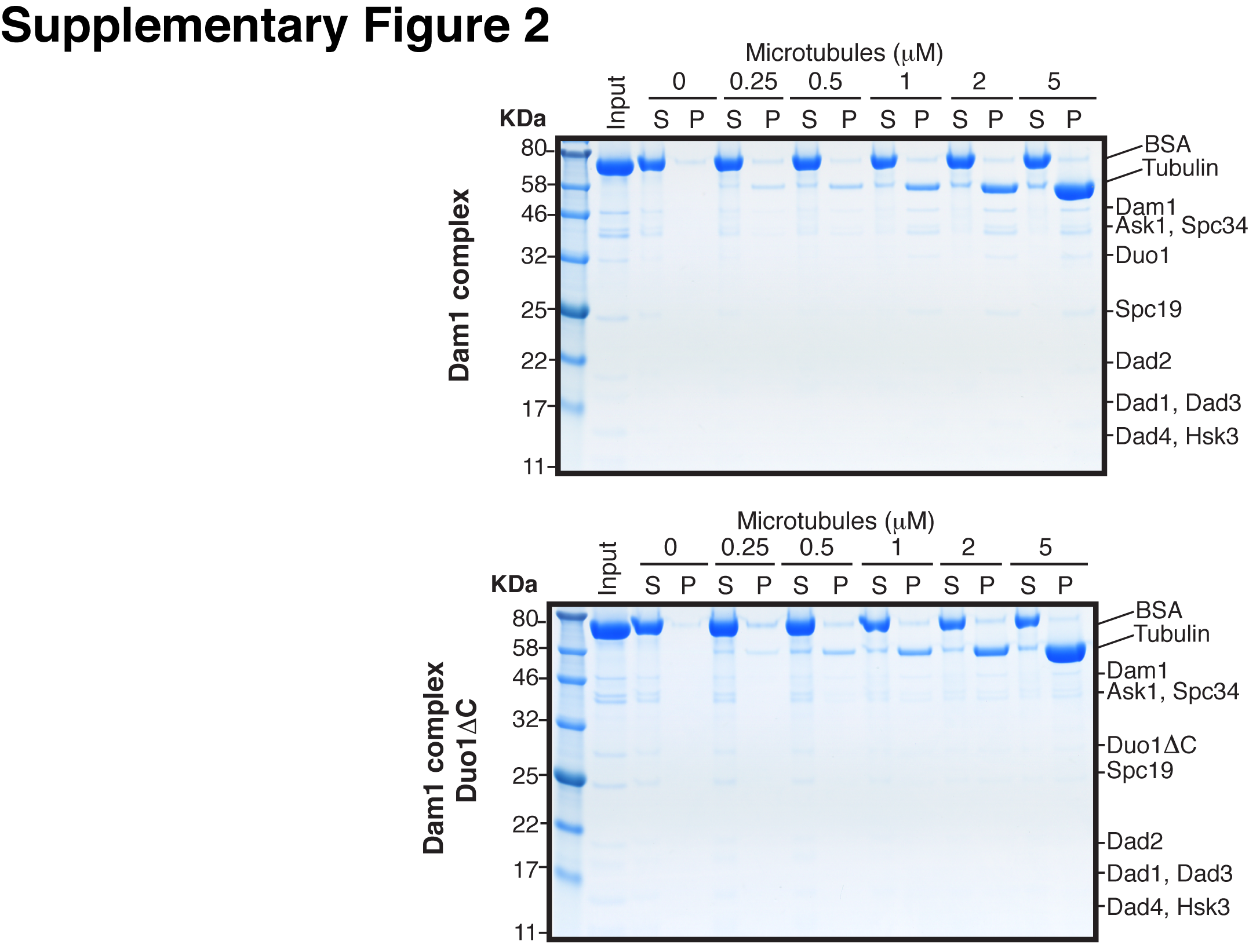

Supplement: Supplementary Figure 2 [file rsob150237supp2.tif]
